# Decoy Receptor 2 as a Cell Cycle Arrest Biomarker for Predicting Renal Recovery Following Acute Kidney Injury

**DOI:** 10.1111/jcmm.70800

**Published:** 2025-08-22

**Authors:** Xiangling Yi, Liming Wang, Xiao‐yue Wang, Yu Fang, Jiarui Liu, Kehong Chen, Ya‐ni He, Jia Chen

**Affiliations:** ^1^ Department of Nephrology, Daping Hospital Army Medical University Chongqing China; ^2^ The Key Laboratory of Precision Diagnosis and Treatment for Kidney Diseases of Chongqing China; ^3^ State Key Laboratory of Trauma, Burn and Combined Injury Army Medical University Chongqing China

**Keywords:** acute kidney injury, biomarker, cell cycle arrest, dcr2, renal recovery

## Abstract

Renal recovery following acute kidney injury (AKI) is a key determinant of long‐term prognosis, while non‐recovery significantly increases the risk of chronic kidney disease and progression to end‐stage renal disease. Although cell cycle arrest is implicated in renal non‐recovery and fibrosis, its association with decoy receptor 2 (DcR2) remains unclear. In this study, we evaluated 139 patients with biopsy‐confirmed AKI, defining renal non‐recovery as a ≥ 50% increase in baseline serum creatinine (Cr) or the initiation of dialysis. Patients were divided into recovery (*n* = 79) and non‐recovery (*n* = 60) groups. Urinary DcR2/creatinine (uDcR2/Cr) levels were significantly higher in non‐recovery cases, with an area under the curve of 0.733 and a diagnostic cut‐off of 378 ng/gCr. Elevated uDcR2/Cr predicted poor renal survival and was independently correlated with non‐recovery. In mouse models of ischemia–reperfusion‐induced AKI, increased urinary and tubular DcR2 levels were also associated with impaired recovery. Proteomics analysis revealed GSK3b enrichment in cell cycle pathways. Functional studies showed that DcR2 mediated cell cycle arrest through GSK3b/cyclin D1 signalling. In conclusion, DcR2 functions as a biomarker of cell cycle arrest and renal recovery, offering both diagnostic and mechanistic insights, and may serve as a potential therapeutic target in AKI.

**Abbreviations**
AKI: acute kidney injury
CDK: cyclin‐dependent kinase
CKD: chronic kidney disease
Cr: creatinine
DcR2: decoy receptor 2
ELISA: enzyme‐linked immunosorbent assay
ESRD: end‐stage renal disease
H/R: hypoxia‐reoxygenation
IGFBP7: insulin‐like growth factor binding protein 7
IRI: ischemia–reperfusion injury
ROC: receiver operating characteristic
RTEC: renal tubular epithelial cell
SASP: senescence‐associated secretory phenotypes
TIMP‐2: tissue inhibitors of metalloproteinase‐2
uNAG: urinary N‐acetyl‐β‐D‐glucosaminidase
α‐SMA: α‐smooth muscle actin

## 1 Introduction

Acute kidney injury (AKI) is a clinical syndrome characterised by a high incidence of kidney damage, leading to structural and functional abnormalities [1]. With advances in diagnosis and recognition, the reported incidence of AKI has increased significantly in recent years. Approximately 10%–15% of hospitalised patients develop AKI, and the incidence exceeds 50% in intensive care units [2]. Despite this prevalence, effective therapeutic interventions for AKI remain limited. Approximately 30% of patients who do not experience renal recovery progress to chronic kidney disease (CKD), with a substantial proportion ultimately advancing to end‐stage renal disease (ESRD) [3]. Even in patients who do recover renal function following AKI, the long‐term risk of developing CKD remains significantly elevated [2, 3]. Consequently, the ability to accurately predict renal recovery after AKI is crucial for guiding clinical decision‐making.

Renal tubular epithelial cells (RTECs) are particularly susceptible to AKI, and cell cycle arrest in these cells contributes to disease pathogenesis and the progression from AKI to CKD [4]. Arrest at the G2–M phase, accompanied by the secretion of profibrotic factors via c‐Jun N‐terminal kinase (JNK) signalling, promotes renal fibrosis following AKI [5]. Moreover, prolonged G1‐phase arrest induces cellular senescence, characterised by the secretion of proinflammatory and profibrotic factors and other components of senescence‐associated secretory phenotypes (SASP), which further impairs renal non‐recovery [6, 7]. Recent single‐cell RNA sequencing has shown that RTECs exhibiting cell cycle arrest predominate after AKI, while proliferative and differentiative cell populations are insufficient, directly contributing to renal non‐recovery [8]. These findings highlight the need to identify the biomarkers of AKI progression and recovery from the perspective of cell cycle arrest.

Decoy receptor 2 (DcR2), a decoy receptor for tumour necrosis factor‐related apoptosis‐inducing ligand (TRAIL), is considered a hallmark of cellular senescence and a marker of irreversible cell cycle arrest [9]. DcR2 is highly expressed in senescent tumour cells and is associated with tumour differentiation and prognosis [9]. A recent study has demonstrated that DcR2 is upregulated in activated hepatic stellate cells and regulates liver fibrosis [10]. Our previous studies found that DcR2 is overexpressed in RTECs in both humans and mouse models of CKD, where its expression correlates with interstitial fibrosis and renal prognosis [11, 12, 13]. However, whether DcR2 expression is associated with renal recovery in AKI remains unclear. Moreover, the extracellular segment of DcR2 is detectable in biological fluids. Serum DcR2 has been proposed as a prognostic marker in prostate cancer [14], and urinary DcR2 (uDcR2) levels have been positively correlated with renal fibrosis in CKD [15, 16]. Whether uDcR2 levels can serve as a prognostic biomarker for renal recovery in AKI has not yet been established.

Cell cycle arrest inhibits RTEC proliferation and promotes fibrosis, impairing recovery after AKI [4]. DcR2 has been identified as a p53 target gene that plays a significant role in tumour cell cycle regulation [17]. In CKD, DcR2‐positive RTECs exhibit G1‐phase arrest, reduced proliferative capacity, and increased SASP expression, hallmarks of senescent cells [12, 15]. Mechanistic studies revealed that DcR2 mediates G1‐phase arrest by interacting with peroxiredoxin 1, promoting RTEC senescence in diabetic nephropathy [12]. Nevertheless, the role of DcR2 in cell cycle arrest and renal recovery during AKI needs to be elucidated.

In this study, we detected urinary and tubular DcR2 levels in patients with biopsy‐confirmed AKI, investigated their association with renal recovery, and validated our findings in a murine model of ischaemia–reperfusion‐induced AKI. Furthermore, we performed mechanistic studies in animal and cellular models to explore the role of DcR2 and its underlying signalling pathways in the context of renal recovery after AKI.

## 2 Methods

### 2.1 Patients

Renal pathology archives from 217 patients diagnosed with AKI between January 2018 and December 2023 at Daping Hospital (Chongqing, China) were reviewed. Patients younger than 18 years and those with anuria, cancer, or urinary tract infections were excluded. Figure S1 presents the flowchart of patient recruitment. Ultimately, 139 patients with biopsy‐confirmed AKI were included in this study. The serum creatinine (Cr) levels and urine output of the included patients met the Kidney Disease: Improving Global Outcomes criteria. All patients were followed for more than 90 d. Renal non‐recovery was defined as either a ≥ 50% increase in baseline serum Cr or the initiation of dialysis due to progression to ESRD within 90 d following AKI diagnosis by renal biopsy [18]. Baseline serum Cr values were obtained 3–6 months before hospital admission or during admission for patients without prior laboratory records [19]. Additionally, 20 normal human kidney samples were obtained from unaffected sections of para‐carcinoma tissues, confirmed to be free of kidney injury on routine pathological examination.

### 2.2 Pathological Scoring of AKI

Renal histology of patients with AKI and mouse models was evaluated using formalin‐fixed sections stained with periodic acid–Schiff (PAS) and Masson's trichrome. The degree of kidney injury was assessed semi‐quantitatively, accounting for both acute and chronic injury. Acute tubulointerstitial injury was graded on a 0–5 scale based on the percentage of tubular necrosis and/or apoptosis, tubular basement membrane denudation, brush border loss, and inflammation (0: no lesion; 1: < 25%; 2: > 25%–50%; 3: > 50–75%; 4: > 75–100%; 5: 100%), as previously described [20]. Chronic tubulointerstitial injury was graded on a 0–4 scale according to the percentage of tubular atrophy and interstitial fibrosis (0: no lesion; 1: < 25%; 2: > 25%–50%; 3: > 50%–75%; 4: > 75%) as previously described [5].

### 2.3 Urinary Biomarker Measurements

Early morning urine samples were collected using standard procedures and centrifuged prior to renal biopsy. Samples were stored at −80°C until analysis. UDcR2 levels were measured using enzyme‐linked immunosorbent assay (ELISA) kits (Hengyuan, Shanghai, China). All samples were diluted 1:4, and all procedures were performed according to the manufacturer's instructions [21]. Urinary levels of tissue inhibitors of metalloproteinase‐2 (TIMP‐2) and insulin‐like growth factor binding protein 7 (IGFBP7) were detected using commercial ELISA kits (Kehbio, Beijing, China) according to the manufacturer's instructions. Urinary N‐acetyl‐β‐D‐glucosaminidase (uNAG) levels were measured using a commercial kit (Aijie Biotechnology, Suzhou, China), as described in our previous studies [15]. To account for variations in urinary concentration, all biomarker levels were normalised to urinary Cr concentrations (mmol/l), as previously described [15].

### 2.4 Immunohistochemical Staining

Renal DcR2 expression was assessed using a two‐step immunohistochemical staining protocol, as described previously [12]. Briefly, sections were deparaffinised and rehydrated. Following antigen retrieval, sections were incubated overnight at 4°C with primary anti‐DcR2 antibody (ab108421; Abcam, Cambridge, UK). A minimum of 10 fields (200× magnification) were randomly selected for the evaluation of DcR2‐positive RTEC staining, which was quantified by calculating the percentage of total RTECs.

### 2.5 Immunofluorescence Staining

Sections were incubated with anti‐DcR2 antibody and anti‐GSK3b antibody (27C10; Cell Signaling Technology), followed by Alexa‐555‐conjugated goat anti‐rabbit antibody (ab150078; Abcam). Additional antibodies used included anti‐α‐SMA (BM0002; Boster Biotechnology, Wuhan, China), anti‐FSP1 (ab218512; Abcam), anti‐collagen I (ab6308; Abcam), anti‐fibronectin (ab6328; Abcam), anti‐p16 (ab54210; Abcam), anti‐p21 (2947S; Cell Signaling Technology), anti‐Ki67 (ab245113; Abcam), anti‐lamin B1 (ab16048; Abcam) and anti‐cyclin D1 (55506S; Cell Signaling Technology). These were followed by Alexa‐488‐conjugated goat anti‐rabbit antibody (ab150078; Abcam) and goat anti‐mouse antibody (ab150117; Abcam). Incubation was performed overnight at 4°C. Sections were co‐stained with DAPI (C1006; Biyuntian Biotechnology, China). Images were acquired using a confocal microscope (Leica, Germany) and analysed using ImageJ software (version 1.37; NIH, Bethesda, MD, USA).

### 2.6 Animal Models

Male C57BL/6J mice (8–10 weeks old) were obtained from the Experimental Animal Center of the Arm Medical University (Chongqing, China). Mice were housed under a standard 12‐h light–dark cycle with free access to food and water. Bilateral ischemia–reperfusion injury (IRI)‐induced AKI models were established as previously described [22]. Briefly, renal ischemia was induced by applying nontraumatic vascular clamps (Aesculap, Biemer 9 mm) to both kidneys for 25 min (moderate IRI) or 35 min (severe IRI) at 37°C, followed by reperfusion upon clamp removal. The maximum observation period for the mouse model was 21 d post‐IRI. Control‐operated mice (Control group) underwent the same surgical procedure, excluding renal pedicle clamping.

### 2.7 Cell Culture and Treatment

Primary mouse RTECs were cultured according to the methods described in our previously published studies [12, 23]. Hypoxia‐reoxygenation (H/R)‐induced cellular models were established by culturing cells under hypoxic conditions (1% O_2_, 94% N_2_ and 5% CO_2_) in FBS‐ and antibiotic‐free medium for 12 h at 37°C, followed by reoxygenation under normoxic conditions (5% CO_2_ and 95% air) [22]. A Polyplus‐transfection system was used to transfect second‐passage RTECs with either control or DcR2‐siRNA plasmids (Obio Technology) [11]. Additionally, cells were pre‐treated with 10 μM AR‐A014418 (A126821; Aladdin Biotechnology, China) prior to H/R exposure. Cells were harvested up to 72 h post‐reoxygenation.

### 2.8 Western Blot (WB) Analysis

WB analysis was performed using renal lysates or cell extracts. The following primary antibodies were used: anti‐GSK3b (27C10; Cell Signaling Technology), anti‐p‐GSK3b^S9^ (SC‐373800; Santa Cruz), anti‐p16 (ab189034; Abcam), anti‐p21 (12D1; Cell Signaling Technology), anti‐cyclin D1 (55506S; Cell Signaling Technology) and anti‐β‐actin (ab198991; Abcam). Band intensities were analysed using Quantity One software (Bio‐Rad, Hercules, CA, USA).

### 2.9 Proteomics and Bioinformatic Analysis

Renal cortex tissue from each sample was lysed and digested with trypsin. Detailed methods for proteomics analysis were described in our previously published study [24]. Proteins with a fold change ≥ 1.5 or ≤ 0.667 and a *t*‐test *p* < 0.05 were defined as differentially expressed proteins (DEPs). Functional analysis of DEPs was performed using gene ontology (GO) pathway derived from the UniProt‐GOA database. GO pathway enrichment results were visualised using the heatmap function.

### 2.10 Statistical Analysis

Data are presented as mean ± standard deviation (SD) for normally distributed variables and as median (interquartile range; IQR) for non‐normally distributed variables. Categorical data are expressed as percentages. Continuous variables were analysed using independent sample t‐tests for normally distributed data; otherwise, the rank‐sum test was applied. Categorical variables were compared using the chi‐squared test. Correlation analyses between uDcR2 levels, the percentage of DcR2‐positive RTECs, and clinical parameters were performed using Pearson's correlation coefficient. Correlation analyses between uDcR2 levels and pathological parameters were performed using Spearman's correlation.

Receiver operating characteristic (ROC) curves were used to analyse the cut‐off values of uDcR2 and uTIMP‐2•IGFBP7 for predicting renal non‐recovery after AKI. ROC analysis of the area under the curve (AUC) was also performed for uDcR2 in combination with estimated glomerular filtration rate (eGFR) and total scores. Additional ROC analyses were performed to assess the predictive value of uDcR2/Cr combined with eGFR and pathological damage of renal function progression in patients with AKI.

The forced introduction method was used to conduct a univariate logistic regression analysis of clinical data. Variables with statistically significant differences in the univariate analysis were included in multifactor logistic regression analyses. Kaplan–Meier survival curves were generated to analyse renal survival time in patients with AKI with different uDcR2/Cr levels. The log‐rank test was used to compare differences between the groups. Multivariate Cox regression analysis was performed to investigate the relationship between uDcR2/Cr and renal recovery in patients with AKI. The stepwise forward method was used to exclude confounding variables and adjust the model. All statistical analyses were conducted using SPSS version 26.0 (SPSS Inc., Chicago, IL, USA). A *p*‐value < 0.05 was considered statistically significant.

## 3 Results

### 3.1 Patient Characteristics

A total of 139 patients with biopsy‐confirmed AKI were enrolled. Their clinical characteristics at biopsy are summarised in Table S1. Patients were categorised into renal recovery (*n* = 79) and renal non‐recovery (*n* = 60) groups based on renal outcomes. In the non‐recovery group, 53 (88.3%) experienced a 50% increase in baseline serum Cr, and 7 (11.7%) required dialysis due to ESRD. No significant differences were observed between the groups in age, sex, body mass index (BMI), AKI causes, urine output, urinary albumin‐to‐creatinine ratio (ACR), or treatment modalities. However, diabetes and CKD were more frequent in the non‐recovery group than in the recovery group. Additionally, significant intergroup differences were noted in serum creatine, eGFR, Cystatin C and pathological scores (acute injury, chronic injury and total scores).

### 3.2 Urinary Biomarker Comparison

The median uDcR2/Cr levels were 213.6 ng/g (IQR: 131.89–331.60) and 419.71 ng/g (IQR: 241.94–621.98) in the recovery and non‐recovery groups, respectively. Similarly, uTIMP‐2•IGFBP7 concentrations were 12.13 (mg/g Cr)^2^/1000 (IQR: 3.74–36.75) and 28.89 (mg/g Cr)^2^/1000 (IQR: 12.00–70.98), respectively. Both markers were significantly elevated in the non‐recovery group. However, uNAG/Cr levels did not differ significantly between the groups (Table S2, Figure S2).

### 3.3 Correlation of uDcR2 With Clinical and Pathological Parameters

Correlation analysis was performed between uDcR2/Cr levels at biopsy and clinical and pathological parameters in patients with AKI. As shown in Table S3, uDcR2/Cr was correlated with chronic injury scores in the non‐recovery group. No correlations were found with age, eGFR, uric acid, BUN, cystatin C, acute injury scores, and total scores in the two groups of patients with recovery and non‐recovery.

### 3.4 Renal Recovery Discrimination of uDcR2 Level.

**FIGURE 1:**
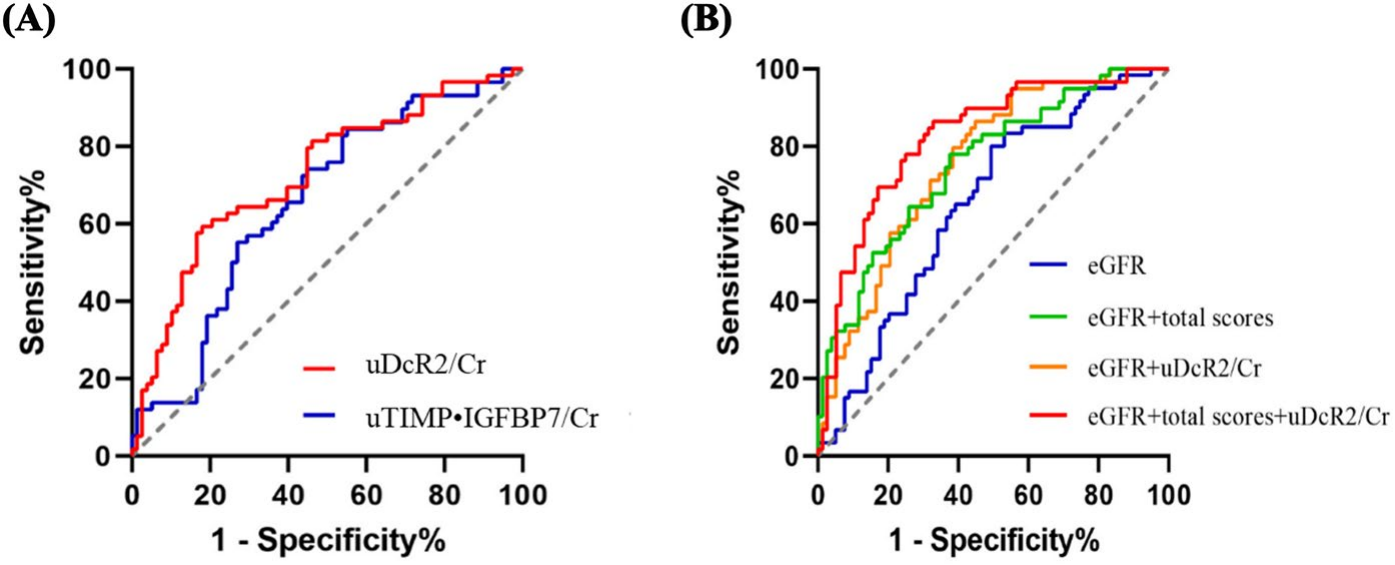
ROC curve analysis of uDcR2/Cr, uTIMP‐2•IGFBP7/Cr (A) and other variables (eGFR and total scores) (B) for discriminating renal recovery in patients with AKI, patients with and without renal recovery.

ROC curve analysis demonstrated that uDcR2 discriminated between recovery and non‐recovery with an AUC of 0.733 (95% confidence interval (CI): 0.646–0.820), higher than that for uTIMP‐2•IGFBP7 AUC = 0.659 (95% CI: 0.566–0.752). At a cut‐off of 378 ng/gCr, uDcR2 showed 60.3% sensitivity and 82.9% specificity (Figure 1A, Table S4A). Adding uDcR2 to eGFR improved the AUC from 0.649 to 0.762. When combined with total pathological scores, the AUC‐ROC improved from 0.753 to 0.824 (Figure 1B, Table S4B).

### 3.5 uDcR2 Was a Risk Factor for Renal Recovery Following AKI

Univariable and multivariable logistic regression analyses were conducted to assess the predictive value of various factors for renal recovery. Diabetes, CKD, cystatin C, uTIMP‐2•IGFBP7 ≥ 14.30 (mg/g Cr)^2^/1000, uDcR2 ≥ 378 ng/g Cr, acute injury, chronic injury, and total scores were identified as risk factors for renal non‐recovery in univariable logistic regression. In multivariable models, both uDcR2 ≥ 378 ng/g Cr and chronic injury scores remained independent risk factors for renal non‐recovery (Table S5).

### 3.6 uDcR2 Predicted Renal Recovery in Patients With AKI

**FIGURE 2:**
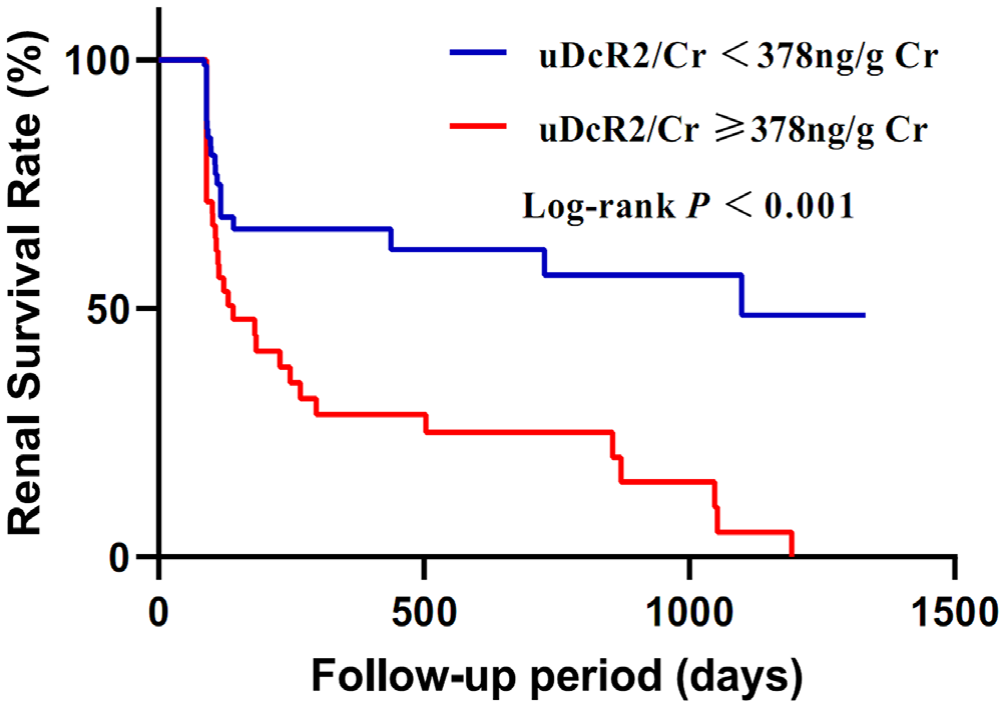
Kaplan–Meier survival curves for renal recovery in different levels of DcR2/Cr.

Kaplan–Meier analysis was performed to analyse renal recovery based on uDcR2/Cr levels. As shown in Figure 2, patients with uDcR2/Cr ≥ 378 ng/gCr exhibited significantly worse renal survival, defined as either a 50% increase in baseline serum Cr or the need for dialysis due to ESRD, than those with uDcR2/Cr < 378 ng/gCr (log‐rank test, *p* < 0.001).

Univariate Cox regression analysis identified several variables associated with renal recovery, including diabetes, cystatin C, uTIMP‐2•IGFBP7 ≥ 14.30 (mg/g Cr)^2^/1000, uDcR2 ≥ 378 ng/gCr, acute injury, chronic injury and total pathological scores (Table S6). However, in multivariate Cox regression, only uDcR2 and chronic injury scores remained significant independent predictors of non‐recovery. To further explore these associations, a series of multivariate Cox proportional hazards models was constructed. Models 1–4 individually assessed the effects of diabetes, eGFR, uTIMP‐2•IGFBP7/Cr and chronic injury scores. Model 5 evaluated whether uDcR2/Cr remained a significant predictor of renal recovery after adjusting for all clinical and pathological variables. In each model, patients with uDcR2/Cr ≥ 378 ng/gCr demonstrated significantly worse renal outcomes than those with lower uDcR2/Cr levels (Model 1: HR = 2.482, *p* < 0.001; Model 2: HR = 2.346, *p* < 0.001; Model 3: HR = 2.809, *p* = 0.009; Model 4: HR = 2.617, *p* < 0.001; Model 5: HR = 2.465, *p* = 0.001; see Table 1).

**TABLE 1:** Multifactorial Cox regression analysis of the associations of uDcR2/Cr level with the renal outcome.

uDcR2/Cr	Uncorrected	Model 1^a^	Model 2^b^	Model 3^c^	Model 4^d^	Model 5^e^
< 378 ng/gCr	1.0 (Ref)	1.0 (Ref)	1.0 (Ref)	1.0 (Ref)	1.0 (Ref)	1.0 (Ref)
≥ 378 ng/gCr	2.659 (1.575–4.488)	2.482 (1.459–4.222)	2.346 (1.382–3.984)	2.809 (1.294–6.095)	2.617 (1.545–4.434)	2.465 (1.124–5.405)
*p*	< 0.001	< 0.001	< 0.001	0.009	< 0.001	0.001

*Note:* Model 1^a^ was adjusted for diabetes; Model 2^b^ was adjusted for eGFR; Model 3^c^ was adjusted for uTIMP‐2•IGFBP7/Cr; Model 4^d^ was adjusted for chronic injury scores; Model 5^e^ was adjusted for history of diabetes, eGFR, uTIMP‐2•IGFBP7/Cr, and chronic injury scores.

### 3.7 Tubular DcR2 Was Associated With Renal Recovery in Patients With AKI

**FIGURE 3:**
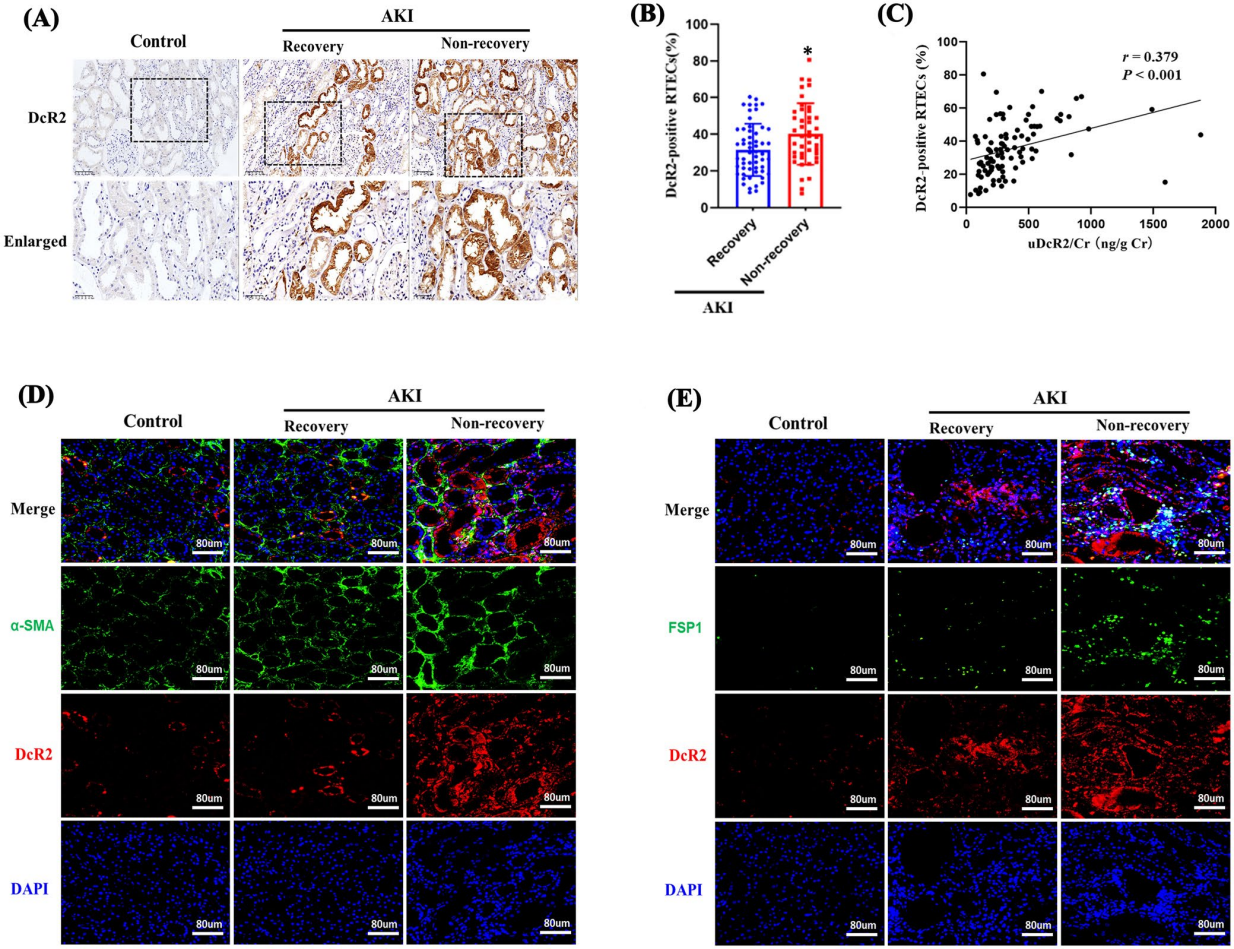
Expression and location of renal DcR2 in patients with AKI. (A) Immuno‐histochemical staining for renal DcR2. The upper scale bar, 100 μm. The lower scale bar, 50 μm. (B) Quantification of DcR2 expression in RTECs. *p < 0.05 vs. renal recovery. (C) Correlation of uDcR2/Cr levels and the percentage of DcR2‐positive RTECs. (D, E) Immunofluorescence double labelling for DcR2 with α‐SMA and FSP1. Scale bar, 80 μm.

Immunohistochemical staining revealed that DcR2 was predominantly localised in renal tubules rather than glomeruli. The proportion of tubular DcR2‐positive cells was significantly higher in patients with renal non‐recovery than in those with recovery (Figure 3A,B). Furthermore, tubular DcR2 expression showed a positive correlation with uDcR2/Cr levels across all patients with AKI (*r* = 0.379, *p* < 0.001; Figure 3C). Fibrotic markers, including α‐smooth muscle actin (α‐SMA) and FSP1, were more prominently expressed in the renal non‐recovery group. These markers were enriched in the vicinity of DcR2‐positive RTECs (Figure 3D,E). Collectively, these findings indicate that elevated tubular DcR2 expression was associated with renal fibrosis and poor recovery outcomes in patients with AKI.

### 3.8 Urinary and Tubular DcR2 Were Associated With Renal Recovery in AKI Mouse Models

**FIGURE 4:**
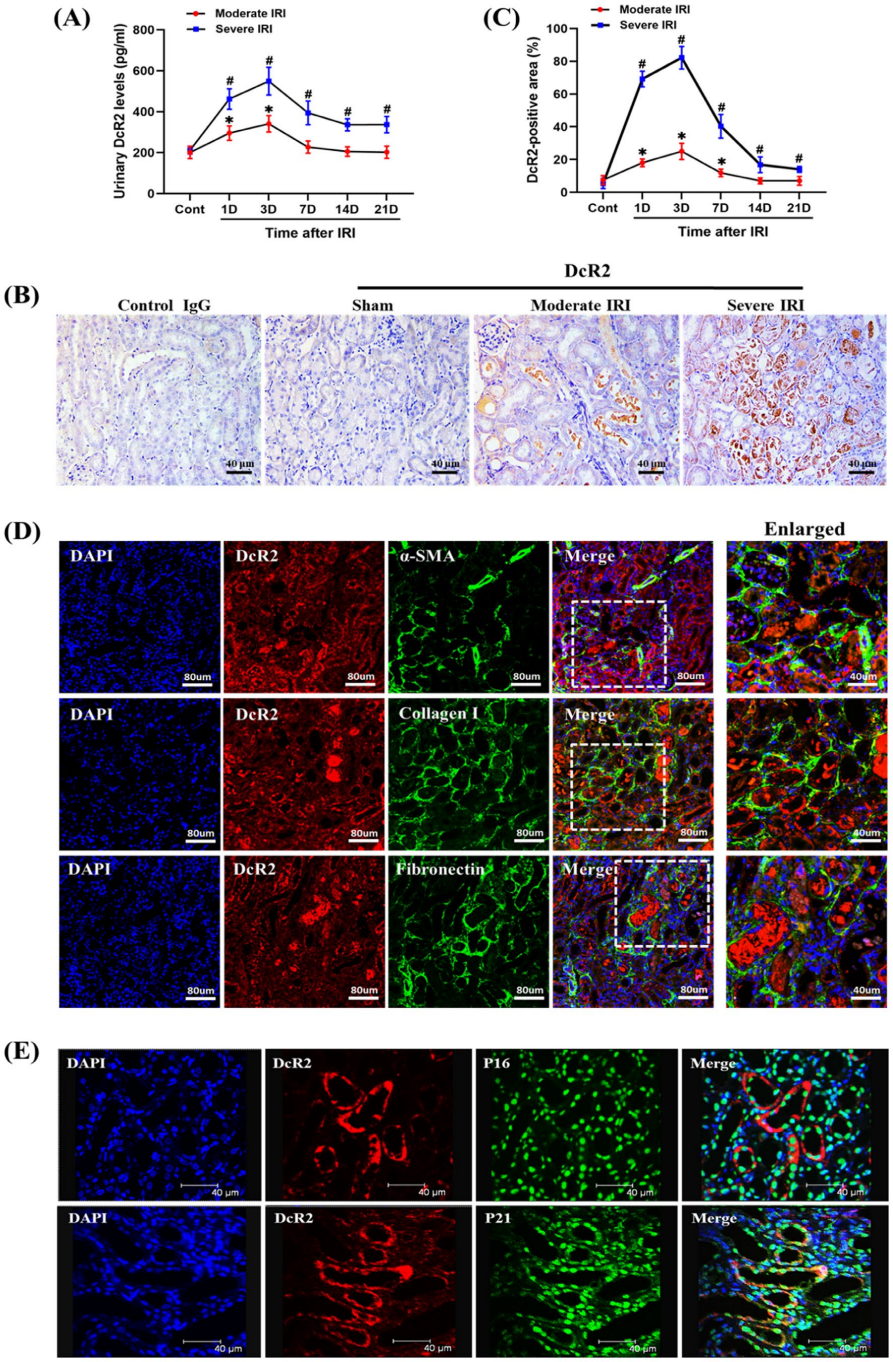
Levels of urinary and tubular DcR2 in AKI mouse models. (A) Levels of urinary DcR2 in moderate and severe IRI. (B, C) Immunohistochemical staining for renal DcR2 and quantification of DcR2‐positive RTECs. Scale bar, 40 μm. (D) Immunofluorescence double labelling for DcR2 with α‐SMA, collagen I, and fibronectin. Scale bar, 40 μm or 80 μm. (E) Immunofluorescence double labelling for DcR2 with p16 and p21. Scale bar, 40 μm. *^/#^ *p* < 0.05 vs. control.

To investigate the association between DcR2 and renal recovery, we developed AKI mouse models representing renal recovery (moderate IRI) and non‐recovery (severe IRI). UDcR2 levels increased and peaked on day 3 following both moderate and severe IRI. With prolonged reperfusion, uDcR2 levels gradually decreased and returned to baseline by day 7 in the moderate IRI (Figure 4A). However, on day 21, uDcR2 levels remained elevated in the severe IRI group compared to those in controls, suggesting an association between uDcR2 and renal non‐recovery. Immunohistochemistry showed that DcR2 was mainly expressed in RTECs and the lumen after moderate and severe IRI (Figure 4B,C). The trend of renal DcR2‐positive staining paralleled that of uDcR2. At 21 d post‐severe IRI, co‐localisation analysis showed DcR2 expression within fibrotic regions positive for α‐SMA, collagen I and fibronectin (Figure 4D). In addition, high expression levels of senescent markers p16 and p21 were observed in DcR2‐positive renal tubular cells (Figure 4E). These results suggest that both urinary and tubular DcR2 were associated with renal non‐recovery in AKI mouse models.

### 3.9 Proteomics Screening of Pathways Involved in Renal Recovery Following AKI

**FIGURE 5:**
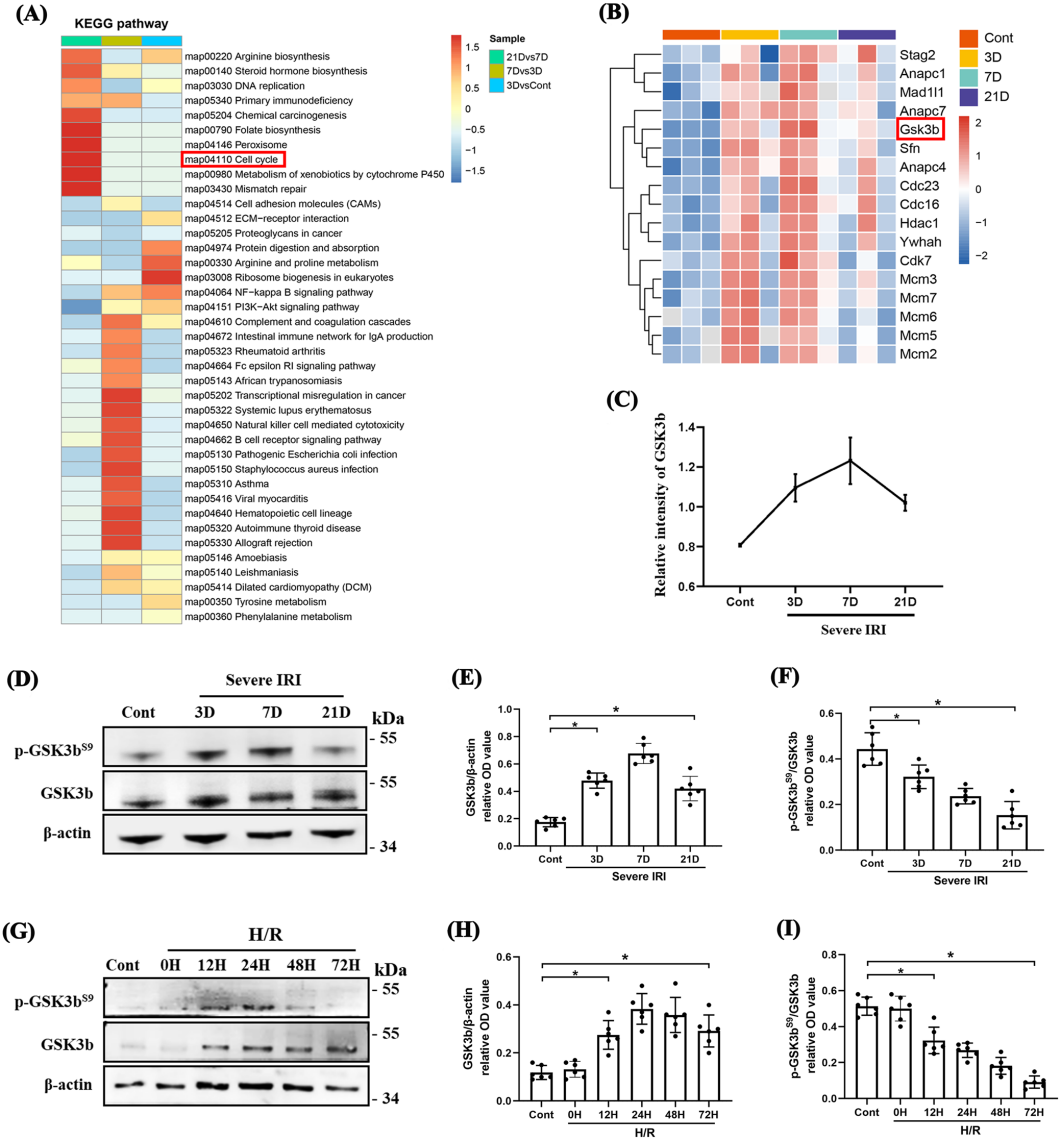
Proteomics identification and validation of cell cycle‐related protein GSK3b expression following AKI. (A) KEGG analysis of different expression proteins (DEPs). (B) Heatmap analysis of cell cycle‐related proteins. (C) Relative expression (quantification intensity) of GSK3b. (D–F) WB analysis for GSK3b and GSK3b^S9^ expression in severe AKI, and their levels are presented relative to those of β‐actin or GSK3b, respectively (*n* = 6). (G–I) WB analysis for GSK3b and GSK3b^S9^ expression after hypoxia reoxygenation (H/R), and quantification of the relative levels to β‐actin or GSK3b in each group, respectively (*n* = 6). Data are expressed as the means ± SDs for each group. *p < 0.05 vs. control.

To explore the mechanism underlying renal non‐recovery after severe IRI, we performed a four‐dimensional proteomic analysis using label‐free quantitative LC–MS/MS. A total of 6158 proteins were quantified, with principal component analysis and differentially expressed DEPs reported in our previous study [24]. Pathway enrichment analysis of DEPs showed several signalling pathways related to tissue repair, including DNA replication, arginine biosynthesis and cell cycle regulation (Figure 5A). The cell cycle plays an important role in biological processes, such as cell proliferation and senescence, which are involved in renal repair after AKI [4, 7]. Therefore, we focused on the cell cycle‐related genes and visualised their enrichment using a heatmap (Figure 5B). GSK3b, a serine/threonine protein kinase known to regulate cell cycle arrest, inhibits tubular regeneration and promotes profibrogenic plasticity [25, 26], emerged as a key molecule. GSK3b expression increased and peaked on day 7 after severe IRI. Although its expression gradually decreased thereafter, it remained elevated compared to controls on day 21 (Figure 5C). These results were further validated both in vivo and in vitro using WB (Figure 5D,E,G,H). Moreover, inhibitory phosphorylation at Ser9 of GSK3b^S9^ and p‐GSK3b^S9^/GSK3b ratio were reduced (Figure 5F,I), suggesting activation of GSK3b after AKI. In both AKI patients and mouse models, GSK3b co‐localised with p16 and p21, which are key inducers of cell cycle arrest. However, GSK3b‐positive RTECs did not express the proliferation marker Ki67 (Figure S3A,B), indicating that these cells had adopted a cell cycle‐arrested phenotype.

### 3.10 DcR2 Mediated Cell Cycle Arrest Through Activation of GSK3b/Cyclin D1 Signalling

**FIGURE 6:**
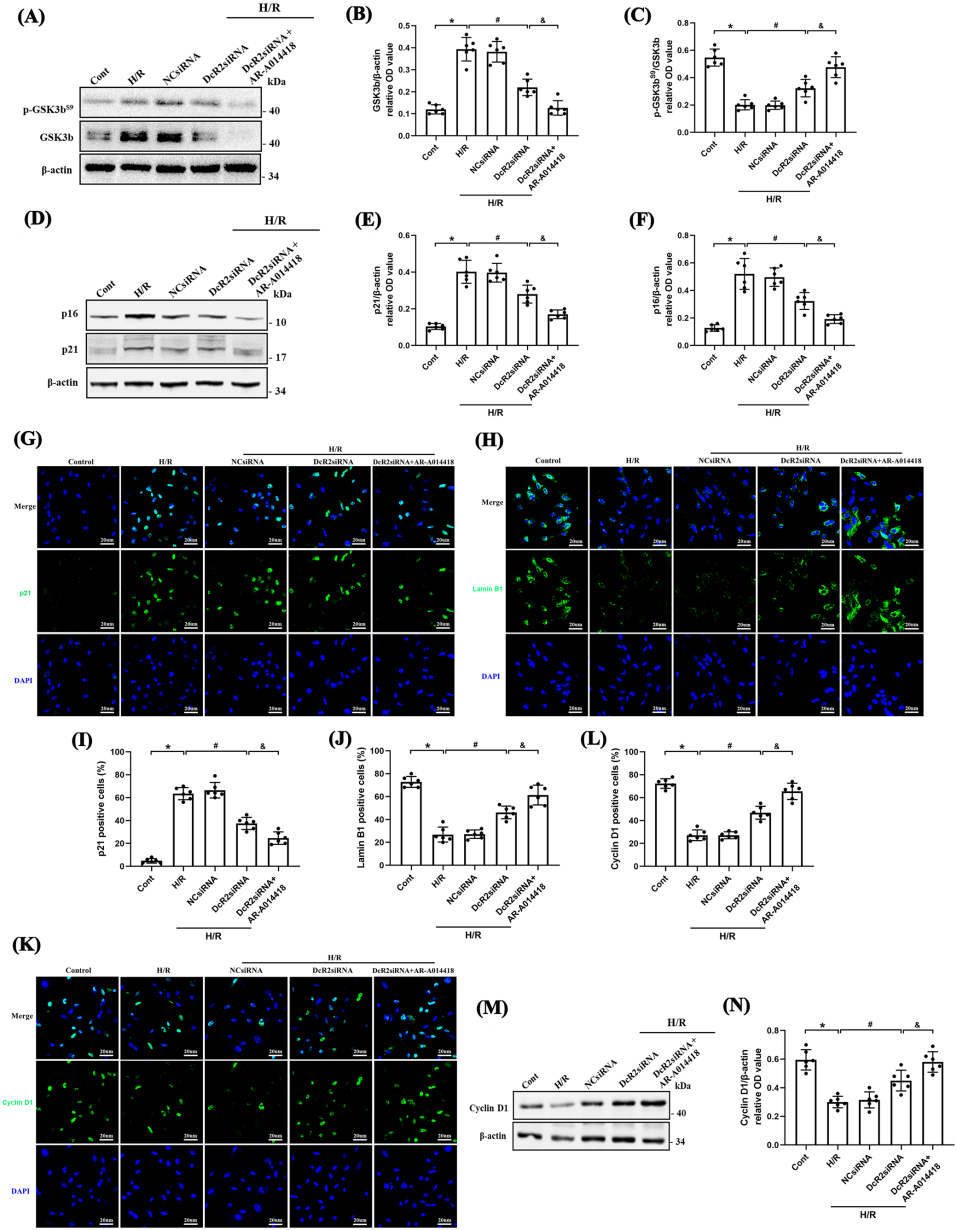
DcR2 Mediates cell cycle arrest through GSK3b/cyclin D1 signalling. (A–C) WB analysis for GSK3b and GSK3b^S9^ expression after downregulation of DcR2 and quantification of the relative levels to β‐actin or GSK3b, respectively (*n* = 6). (D–F) WB analysis for p16 and p21 expression and quantification of the relative levels to β‐actin in each group (*n* = 6). (G–L) Immunofluorescence analysis of the expression of p21, Lamin B1, and cyclin D1 and the percentage of positive cells. Scale bar, 20 μm. (M, N) WB analysis for cyclin D1 expression, and quantification of the relative levels to β‐actin in each group (*n* = 6). *p < 0.05 vs. control, ^#^ *p* < 0.05 vs. H/R, ^&^ *p* < 0.05 vs. DcR2siRNA.

To elucidate the mechanism of DcR2‐mediated cell cycle arrest, we regulated DcR2 expression in H/R‐induced primary RTECs. As shown in Figure 6A–C, DcR2 downregulation decreased H/R‐induced GSK3b expression and increased the p‐GSK3b^S9^/GSK3b ratio, indicating inhibited GSK3b activity. Concurrently, DcR2 downregulation inhibited p16 and p21 expression, as determined by WB, and immunofluorescence staining revealed reduced p21 expression and increased Lamin B1 expression (Figure 6D–J). Pharmacological inhibition of GSK3b using AR‐A014418 further elevated p‐GSK3b^S9^/GSK3b ratios, while reducing p16 and p21 and increasing Lamin B1 expression, corroborating the suppressive effect of GSK3b inhibition on senescence‐related signalling. In addition, we observed that both DcR2 and GSK3b regulated cyclin D1 expression (Figure 6K–N), a key protein involved in cell progression. Collectively, these findings indicate that DcR2 mediates cell cycle arrest via activation of GSK3b/cyclin D1 signalling.

## 4 Discussion

AKI is a prevalent clinical syndrome associated with high mortality, morbidity and healthcare costs, particularly in critically ill patients. Even among those who initially recover renal function, the long‐term risk of progression to CKD or ESRD remains substantial [2]. In this study, we demonstrated that uDcR2 was an independent predictor of renal recovery in patients with AKI. Moreover, urinary and tubular DcR2 levels were associated with renal outcomes in IRI‐induced AKI mouse models. Proteomic screening and validation studies demonstrated that DcR2 mediated tubular cell cycle arrest through the GSK3b/cyclin D1 signalling axis, contributing to renal fibrosis following AKI.

Current biomarker research in AKI primarily focuses on the early diagnosis of utilising markers, such as TIMP‐2, IGFBP7, Kim‐1, NGAL and CXCL9 [27]. The Food and Drug Administration‐approved TIMP‐2•IGFBP7 has shown value in predicting early diagnosis of AKI; however, its utility in predicting renal recovery remains unclear. Previous studies have found that patients with AKI with TIMP‐2•IGFBP7 > 2.0 had an increased risk of death or dialysis after 9 months [28]. Although TIMP‐2•IGFBP7 has also been associated with adverse outcomes in patients, its sensitivity and specificity in predicting renal recovery need improvement [18, 19]. In our study, while uTIMP‐2•IGFBP7 levels were higher in patients who failed to recover, uDcR2 outperformed them in predictive accuracy.

DcR2, a type II transmembrane protein, undergoes extracellular segment cleavage [29]. Our previous studies demonstrated a correlation among uDcR2 levels, renal function and interstitial fibrosis in CKD [15, 16]. In this study, uDcR2 levels were significantly higher in patients with renal non‐recovery than in those who recovered. ROC curve analysis showed an AUC of 0.733 for uDcR2, surpassing that for uTIMP‐2•IGFBP7. Combining uDcR2 with clinical indicators, such as estimated eGFR and histopathological scores, improved the prediction of renal recovery. Using a cut‐off (≥ 378 ng/gCr), uDcR2 was identified as an independent risk factor for non‐recovery. Patients with uDcR2/Cr ≥ 378 ng/gCr exhibited significantly lower renal survival. Previous studies have identified diabetes history, baseline eGFR, and chronic injury scores as predictors of renal recovery after AKI [2, 18]. Multivariate Cox regression models demonstrated that uDcR2/Cr predicted renal recovery independent of these clinical and pathological variables. These results indicate that uDcR2 is a superior and independent biomarker for evaluating renal recovery following AKI.

To investigate the source of uDcR2, we analysed renal biopsy tissue and found that DcR2 was predominantly expressed in renal tubules, with minimal expression in the glomeruli, consistent with previous CKD findings [13, 15]. Previous studies have shown that DcR2 upregulation contributes to immune surveillance of senescent cells and promotes liver fibrosis [10]. In diabetic nephropathy, tubular DcR2 has been implicated in mediating senescence‐associated secretory and apoptosis‐resistant phenotypes, exacerbating renal fibrosis [11, 12]. In our study, patients with renal non‐recovery exhibited a higher proportion of DcR2‐positive tubular cells than those who recovered. Notably, DcR2 expression was predominantly localised in fibrotic regions characterised by α‐SMA, collagen I and fibronectin expression. Similar patterns were observed in IRI AKI mouse models. These findings suggest that tubular DcR2 is correlated with renal non‐recovery after AKI and may contribute to the progression of renal fibrosis, a key driver of structural damage and dysfunction [30].

Quantitative proteomic and bioinformatic analyses revealed that several pathways, including cell cycle regulation, arginine biosynthesis, and other metabolism‐related signalling cascades, are involved in AKI. The cell cycle plays a critical role not only in renal repair but also in the progression of renal fibrosis. Therapeutic strategies to prevent renal fibrosis include targeting epithelial cell cycle arrest in G2‐M phase, which mediates fibrosis via JNK signalling, and eliminating G2/M‐arrested tubular cells through downstream regulatory pathways [5]. Additionally, arrest at the G1/S phase promotes cellular senescence and the secretion of SASP factors, which contribute to fibrotic progression [6]. In this study, GSK3b was enriched in cell cycle‐related pathways. GSK3b plays a key role in kidney injury across various diseases, including AKI, diabetic nephropathy and renal ageing [25, 31, 32]. Notably, GSK3b expression peaked on day 7 after severe IRI, accompanied by reduced inhibitory phosphorylation at Ser9 and decreased p‐GSK3b^S9^/GSK3b ratios, indicating GSK3b activation during the course of injury.

Previous studies have shown that renal GSK3b overactivity increases the phosphorylation of p16 and p53, which are required for cell cycle arrest and senescence promotion. As substrates of GSK3b, p16 and p53 drive podocyte senescence [32]. In both patients and mouse models with AKI, we found that GSK3b was co‐localised with p16 and p21, but not with Ki67, indicating that GSK3b‐positive RTECs exhibited a phenotype of cell cycle arrest. After downregulation of DcR2 in vitro, GSK3b expression decreased, and the p‐GSK3b^S9^/GSK3b ratio increased, suggesting that DcR2 regulated GSK3b activity. DcR2 downregulation inhibited p16 and p21 expression and enhanced Lamin B1 expression, demonstrating that DcR2 regulated cell cycle arrest after AKI. Subsequent intervention experiments with the GSK3b inhibitor AR‐A014418 confirmed that DcR2 regulated cell cycle arrest via GSK3b.

The cell cycle is controlled by cyclin‐dependent kinase (CDK) complexes. CDKs do not exhibit kinase activity on their own; however, these kinases require their binding partners, cyclins, for substrate phosphorylation. Cyclin D1 binds to CDK4 or CDK6 and CDK2 and sequentially phosphorylates the retinoblastoma protein (Rb), facilitating the G1 to S phase transition. Previous studies have reported that cyclin D1 is phosphorylated on Thr^286^ by GSK3b, triggering its nuclear export, ubiquitination, and subsequent degradation, thereby regulating cell cycle arrest [33, 34]. Moreover, GSK3b plays an important role in cyclin D1 expression by regulating both its mRNA transcription and protein levels [34]. Consistent with this phenomenon, in this study, GSK3b was found to regulate cyclin D1 expression, further indicating that DcR2 mediates cell cycle arrest through GSK3b/cyclin D1 signalling.

However, this study has some limitations. First, as a retrospective cohort study, the findings require validation in prospective studies. Second, the sample size was relatively small, and all patients were enrolled from a single centre, which may limit the generalisability of the results. Although uDcR2 levels were associated with renal non‐recovery in this cohort, larger multicentre studies are necessary to validate these findings. Third, due to the retrospective nature of the study, urine samples were not consistently collected during the outpatient follow‐up, and uDcR2 levels at follow‐up endpoints were not available. Nonetheless, the results suggest that uDcR2 levels at the time of renal biopsy remain valuable for predicting renal outcomes.

## 5 Conclusions

Urinary DcR2 levels and tubular DcR2 expression were associated with renal recovery in both patients and mouse models of AKI. As a noninvasive biomarker, uDcR2 independently predicted renal recovery, regardless of clinical and pathological variables. Future large‐scale, multicentre studies are warranted to further validate the predictive value of uDcR2. Mechanistically, DcR2 mediated tubular cell cycle arrest through GSK3b/cyclin D1 signalling, contributing to renal non‐recovery and fibrosis after AKI. These findings suggest that DcR2 may serve as a potential therapeutic target for AKI.

## Author Contributions

**Xiangling Yi:** formal analysis (equal), writing – review and editing (equal). **Liming Wang:** formal analysis (equal). **Xiao‐yue Wang:** validation (equal). **Yu Fang:** validation (equal). **Jiarui Liu:** data curation (equal), formal analysis (equal). **Kehong Chen:** funding acquisition (equal), investigation (equal), supervision (equal). **Ya‐ni He:** funding acquisition (equal), supervision (equal), writing – review and editing (equal). **Jia Chen:** funding acquisition (equal), project administration (equal), writing – original draft (equal), writing – review and editing (equal).

## Ethics Statement

This study was conducted in accordance with the principles of the Declaration of Helsinki. The study protocol was approved by the Human Ethics and Protocol Review Committee of the Army Medical University (NO. 2022–113), and written informed consent was obtained from all patients. The animal experiments were approved by the Ethics Committee of the Army Medical University (NO. AMUWEC20210905).

## Consent

The authors have nothing to report.

## Conflicts of Interest

The authors declare no conflicts of interest.

## Supporting information


**Table S1:** Baseline characteristics between patients with AKI, with and without renal recovery.
**Table S2:** Urinary biomarker values grouped according to the renal recovery status.
**Table S3:** Correlation of uDcR2 with clinical and pathological parameters.
**Table S4A:** Urinary biomarkers for predicting renal non‐recovery from AKI.
**Table S4B:** Urinary biomarkers and combination models for predicting renal non‐recovery from AKI.
**Table S5:** Univariable and multivariable logistic regression analyses for risk factors for renal non‐recovery.
**Table S6:** Predictive factors for renal non‐recovery via univariate and multivariate Cox regression analyses.
**Figure S1:** Flowchart of patient recruitment.
**Figure S2:** Levels of urinary biomarkers in patients with AKI in the recovery and non‐recovery groups. (A) uDcR2/Cr levels. (B) uTIMP‐2•IGFBP7 levels. (C) uNAG/Cr levels.
**Figure S3:** Immunofluorescence double labelling for GSK3b with p16, p21 and Ki67 in patients with AKI (A) and mouse models (B). Scale bar, 40 μm.

## Data Availability

Authors declared that all data supporting the findings are available within the paper. The raw data that support the findings are available from the corresponding author upon reasonable request.

## References

[1] R. Bellomo , J. A. Kellum , and C. Ronco , “Acute Kidney Injury,” Lancet 380, no. 9843 (2012): 756–766, 10.1016/S0140-6736(11)61454-2. 22617274

[2] C. Ronco , R. Bellomo , and J. A. Kellum , “Acute Kidney Injury,” Lancet 394, no. 10212 (2019): 1949–1964, 10.1016/S0140-6736(19)32563-2. 31777389

[3] E. A. J. Hoste , J. A. Kellum , N. M. Selby , et al., “Global Epidemiology and Outcomes of Acute Kidney Injury,” Nature Reviews. Nephrology 14, no. 10 (2018): 607–625, 10.1038/s41581-018-0052-0. 30135570

[4] W. G. Wang , W. X. Sun , B. S. Gao , X. Lian , and H. L. Zhou , “Cell Cycle Arrest as a Therapeutic Target of Acute Kidney Injury,” Current Protein & Peptide Science 18, no. 12 (2017): 1224–1231, 10.2174/1389203717666160915162238. 27634440

[5] L. Yang , T. Y. Besschetnova , C. R. Brooks , J. V. Shah , and J. V. Bonventre , “Epithelial Cell Cycle Arrest in G2/M Mediates Kidney Fibrosis After Injury,” Nature Medicine 16, no. 5 (2010): 535–543, 10.1038/nm.2144. PMC3928013 20436483

[6] J. Chen , H. Zhang , X. Yi , et al., “Cellular Senescence of Renal Tubular Epithelial Cells in Acute Kidney Injury,” Cell Death Discovery 10, no. 1 (2024): 62, 10.1038/s41420-024-01831-9. 38316761 PMC10844256

[7] W. Huang , L. J. Hickson , A. Eirin , J. L. Kirkland , and L. O. Lerman , “Cellular Senescence: The Good, the Bad and the Unknown,” Nature Reviews Nephrology 18, no. Oct (2022): 611–627, 10.1038/s41581-022-00601-z. 35922662 PMC9362342

[8] L. M. S. Gerhardt , J. Liu , K. Koppitch , P. E. Cippa , and A. P. McMahon , “Single‐Nuclear Transcriptomics Reveals Diversity of Proximal Tubule Cell States in a Dynamic Response to Acute Kidney Injury,” Proceedings of the National Academy of Sciences of The United States of America 118, no. 27 (2021): 1‐11. 10.1073/pnas.2026684118. PMC8271768 34183416

[9] M. Collado , J. Gil , A. Efeyan , et al., “Tumour Biology: Senescence in Premalignant Tumours,” Nature 436, no. 7051 (2005): 642, 10.1038/436642a. 16079833

[10] A. Sagiv , A. Biran , M. Yon , J. Simon , S. W. Lowe , and V. Krizhanovsky , “Granule Exocytosis Mediates Immune Surveillance of Senescent Cells,” Oncogene 32, no. 15 (2013): 1971–1977, 10.1038/onc.2012.206. 22751116 PMC3630483

[11] J. Chen , K. H. Chen , L. M. Wang , J. Luo , Q. Y. Zheng , and Y. N. He , “Decoy Receptor 2 Mediates the Apoptosis‐Resistant Phenotype of Senescent Renal Tubular Cells and Accelerates Renal Fibrosis in Diabetic Nephropathy,” Cell Death & Disease 13, no. 6 (2022): 522, 10.1038/s41419-022-04972-w. 35661704 PMC9166763

[12] C. Jia , C. Ke‐Hong , X. Fei , et al., “Decoy Receptor 2 Mediation of the Senescent Phenotype of Tubular Cells by Interacting With Peroxiredoxin 1 Presents a Novel Mechanism of Renal Fibrosis in Diabetic Nephropathy,” Kidney International 98, no. 3 (2020): 645–662, 10.1016/j.kint.2020.03.026. 32739204

[13] H. Dai , W. Hu , L. Lin , L. Wang , J. Chen , and Y. He , “Tubular Decoy Receptor 2 as a Predictor of Prognosis in Patients With Immunoglobulin A Nephropathy,” Clinical Kidney Journal 14, no. 5 (2021): 1458–1468, 10.1093/ckj/sfaa257. 33959273 PMC8087134

[14] I. J. Sanchez‐Lazaro , L. Almenar‐Bonet , A. Romero‐Pelechano , et al., “Serum Markers of Apoptosis in the Early Period of Heart Transplantation,” Biomarkers 17, no. 3 (2012): 254–260, 10.3109/1354750X.2012.664168. 22435528

[15] J. Chen , W. W. Zhang , K. H. Chen , et al., “Urinary DcR2 Is a Novel Biomarker for Tubulointerstitial Injury in Patients With Diabetic Nephropathy,” American Journal of Physiology. Renal Physiology 313, no. 2 (2017): F273–F281, 10.1152/ajprenal.00689.2016. 28356293

[16] J. Chen , W. Hu , F. Xiao , et al., “DCR2, a Cellular Senescent Molecule, Is a Novel Marker for Assessing Tubulointerstitial Fibrosis in Patients With Immunoglobulin A Nephropathy,” Kidney & Blood Pressure Research 44, no. 5 (2019): 1063–1074, 10.1159/000502233. 31487717

[17] R. D. Meng , E. R. McDonald, 3rd , M. S. Sheikh , A. J. Fornace, Jr. , and W. S. El‐Deiry , “The TRAIL Decoy Receptor TRUNDD (DcR2, TRAIL‐R4) is Induced by Adenovirus‐p53 Overexpression and Can Delay TRAIL‐, p53‐, and KILLER/DR5‐Dependent Colon Cancer Apoptosis,” Molecular Therapy 1, no. 2 (2000): 130–144, 10.1006/mthe.2000.0025. 10933923

[18] H. M. Jia , L. Cheng , Y. B. Weng , et al., “Cell Cycle Arrest Biomarkers for Predicting Renal Recovery From Acute Kidney Injury: A Prospective Validation Study,” Annals of Intensive Care 12, no. 1 (2022): 14, 10.1186/s13613-022-00989-8. 35150348 PMC8840946

[19] Y. Xie , G. Ankawi , B. Yang , et al., “Tissue Inhibitor Metalloproteinase‐2 (TIMP‐2) * IGF‐Binding Protein‐7 (IGFBP7) Levels Are Associated With Adverse Outcomes in Patients in the Intensive Care Unit With Acute Kidney Injury,” Kidney International 95, no. 6 (2019): 1486–1493, 10.1016/j.kint.2019.01.020. 30982674

[20] L. Yang , C. R. Brooks , S. Xiao , et al., “KIM‐1‐Mediated Phagocytosis Reduces Acute Injury to the Kidney,” Journal of Clinical Investigation 125, no. 4 (2015): 1620–1636, 10.1172/JCI75417. 25751064 PMC4396492

[21] J. Chen , K. H. Chen , L. M. Wang , et al., “High‐Dose HOOK Effect in Urinary DcR2 Assay in Patients With Chronic Kidney Disease,” Clinical Biochemistry 58 (2018): 32–36, 10.1016/j.clinbiochem.2018.06.001. 29879421

[22] J. Chen , H. Lu , X. Wang , et al., “VNN1 Contributes to the Acute Kidney Injury‐Chronic Kidney Disease Transition by Promoting Cellular Senescence via Affecting RB1 Expression,” FASEB Journal 36, no. 9 (2022): e22472, 10.1096/fj.202200496RR. 35959877

[23] J. Chen , K. H. Chen , B. Q. Fu , et al., “Isolation and Identification of Senescent Renal Tubular Epithelial Cells Using Immunomagnetic Beads Based on DcR2,” Experimental Gerontology 95 (2017): 116–127, 10.1016/j.exger.2017.04.008. 28461078

[24] J. Chen , Q. Y. Zheng , L. M. Wang , J. Luo , K. H. Chen , and Y. N. He , “Proteomics Reveals Defective Peroxisomal Fatty Acid Oxidation During the Progression of Acute Kidney Injury and Repair,” Heliyon 9, no. 7 (2023): e18134, 10.1016/j.heliyon.2023.e18134. 37539197 PMC10395357

[25] B. Chen , P. Wang , X. Liang , et al., “Permissive Effect of GSK3beta on Profibrogenic Plasticity of Renal Tubular Cells in Progressive Chronic Kidney Disease,” Cell Death Dis 12, no. 5 (2021): 432, 10.1038/s41419-021-03709-5. 33931588 PMC8087712

[26] S. Sinha , N. Dwivedi , J. Woodgett , et al., “Glycogen Synthase Kinase‐3β Inhibits Tubular Regeneration in Acute Kidney Injury by a FoxM1‐Dependent Mechanism,” FASEB Journal 34, no. 10 (2020): 13597–13608, 10.1096/fj.202000526RR. 32813289 PMC7722032

[27] Y. Wen and C. R. Parikh , “Current Concepts and Advances in Biomarkers of Acute Kidney Injury,” Critical Reviews in Clinical Laboratory Sciences 58, no. 5 (2021): 354–368, 10.1080/10408363.2021.1879000. 33556265

[28] M. Joannidis , L. G. Forni , M. Haase , et al., “Use of Cell Cycle Arrest Biomarkers in Conjunction With Classical Markers of Acute Kidney Injury,” Critical Care Medicine 47, no. 10 (2019): e820–e826, 10.1097/CCM.0000000000003907. 31343478 PMC6750148

[29] C. Lorz , A. Benito , A. C. Ucero , B. Santamaria , and A. Ortiz , “Trail and Kidney Disease,” Front Biosci (Landmark Ed) 14, no. 10 (2009): 3740–3749, 10.2741/3485. 19273307

[30] A. Niculae , M. E. Gherghina , I. Peride , M. Tiglis , A. M. Nechita , and I. A. Checherita , “Pathway From Acute Kidney Injury to Chronic Kidney Disease: Molecules Involved in Renal Fibrosis,” International Journal of Molecular Sciences 24, no. 18 (2023): 14019, 10.3390/ijms241814019. 37762322 PMC10531003

[31] L. L. Liang , M. F. He , P. P. Zhou , S. K. Pan , D. W. Liu , and Z. S. Liu , “GSK3beta: A Ray of Hope for the Treatment of Diabetic Kidney Disease,” FASEB J 38, no. 3 (2024): e23458, 10.1096/fj.202302160R. 38315453

[32] Y. Fang , B. Chen , Z. Liu , et al., “Age‐Related GSK3beta Overexpression Drives Podocyte Senescence and Glomerular Aging,” J Clin Invest 132, no. 4 (2022):1‐16. 10.1172/JCI141848. PMC8843754 35166234

[33] S. L. Liu , Z. Liu , L. D. Zhang , et al., “GSK3beta‐Dependent Cyclin D1 and Cyclin E1 Degradation Is Indispensable for NVP‐BEZ235 Induced G0/G1 Arrest in Neuroblastoma Cells,” Cell Cycle 16, no. 24 (2017): 2386–2395, 10.1080/15384101.2017.1383577. 28980866 PMC5788431

[34] F. Takahashi‐Yanaga and T. Sasaguri , “GSK‐3beta Regulates Cyclin D1 Expression: A New Target for Chemotherapy,” Cellular Signalling 20, no. 4 (2008): 581–589, 10.1016/j.cellsig.2007.10.018. 18023328

