# Supplementary material for: Decoy Receptor 2 as a Cell Cycle Arrest Biomarker for Predicting Renal Recovery Following Acute Kidney Injury
Source: J Cell Mol Med. 2025 Aug 22;29(16):e70800. doi: 10.1111/jcmm.70800 (PMC12372981; doi:10.1111/jcmm.70800)
Supplement: Supplementary file 1 — Table S1: Baseline characteristics between patients with AKI, with and without renal recovery. Table S2: Urinary biomarker values grouped according to the renal recovery status. Table S3: Correlation of uDcR2 with clinical and pathological parameters. Table S4A: Urinary biomarkers for predicting renal non‐recovery from AKI. Table S4B: Urinary biomarkers and combination models for predicting renal non‐recovery from AKI. Table S5: Univariable and multivariable logistic regression analyses for risk factors for renal non‐recovery. Table S6: Predictive factors for renal non‐recovery via univariate and multivariate Cox regression analyses. Figure S1: Flowchart of patient recruitment. Figure S2: Levels of urinary biomarkers in patients with AKI in the recovery and non‐recovery groups. (A) uDcR2/Cr levels. (B) uTIMP‐2•IGFBP7 levels. (C) uNAG/Cr levels. Figure S3: Immunofluorescence double labelling for GSK3b with p16, p21 and Ki67 in patients with AKI (A) and mouse models (B). Scale bar, 40 μm. [file JCMM-29-e70800-s001.docx]

**Supplementary Table 1. Baseline characteristics between AKI patients with and without renal recovery**

| **Variables** | **Total** | **Renal recovery** | **Renal non-recovery** | ***P* value** |
| --- | --- | --- | --- | --- |
| Numbers | 139 | 79 | 60 |  |
| Age (years) | 50±17 | 48±18 | 53±17 | 0.122 |
| Sex, M/F | 81/58 | 43/36 | 38/22 | 0.292 |
| BMI (kg/m^2^) | 23.81 (20.77, 25.84) | 23.83 (20.74, 26.61) | 23.55 (20.77, 25.03) | 0.419 |
| **Comorbidities** |  |  |  |  |
| Hypertension, n (%) | 40 (28.8) | 22 (27.8) | 18 (30.0) | 0.781 |
| Diabetes, n (%) | 19 (13.7) | 6 (7.6) | 13 (21.7)^*^ | 0.017 |
| CKD, n (%) | 89 (64.0) | 44 (55.7) | 45 (75.0)^*^ | 0.019 |
| **Causes of AKI** |  |  |  |  |
| Medication, n (%) | 53 (38.1) | 27(34.2) | 26 (43.3) | 0.271 |
| Infection, n (%) | 19 (13.7) | 14 (17.7) | 5 (8.3) | 0.111 |
| Autoimmunity, n (%) | 37 (26.6) | 19 (24.1) | 18 (30.0) | 0.432 |
| Others, n (%) | 30 (21.6) | 19 (24.1) | 11 (18.3) | 0.417 |
| **Laboratory parameters** |  |  |  |  |
| SCr (umol/L) | 218.70(143.05, 397.10) | 173.00(115.75, 307.98) | 239.85(182.10, 508.65) | 0.005 |
| eGFR (mL/min/1.73 m^2^) | 26.06 (12.67, 46.38) | 34.38 (15.93, 51.77) | 22.72 (10.18, 32.87)^*^ | 0.003 |
| Uric acid (mmol/L) | 453.00±196.93 | 465.51±221.35 | 436.53±159.54 | 0.392 |
| BUN (mmol/L) | 11.53 (7.87, 18.12) | 10.70 (7.07, 16.41) | 12.16 (9.05, 19.44) | 0.106 |
| Cystatin C (mg/L) | 2.80±1.50 | 2.31±1.05 | 3.40±1.75^*^ | 0.001 |
| Urine output (ml) | 709 (458, 926) | 645 (473, 904) | 650 (406, 946) | 0.735 |
| Urinary ACR (mg/g*Cr) | 175.70 (40.76, 1822.70) | 198.47 (51.81, 3305.02) | 688.89 (159.14, 2158.58) | 0.736 |
| **Pathological scores** |  |  |  |  |
| Acute injury scores | 2.36±1.07 | 2.07±1.03 | 2.74±1.02^*^ | <0.001 |
| Chronic injury scores | 1.64±1.03 | 1.24±0.77 | 2.14±1.10^*^ | <0.001 |
| Total scores | 4.00±1.87 | 3.31±1.60 | 4.88±1.83^*^ | <0.001 |
| **Treatment** |  |  |  |  |
| Corticosteroid, n (%) | 87 (62.6) | 47 (59.5) | 40 (66.7) | 0.387 |
| RRT, n (%) | 23 (16.5) | 11 (11.9) | 12 (20.0) | 0.340 |
| **Renal Outcome**, n (%) |  |  |  |  |
| 50% increase of SCr | 53 (38.1) |  | 53 (88.3) |  |
| Dialysis | 7 (5.0) |  | 7 (11.7) |  |

BMI, body mass index; CKD, chronic kidney disease; SCr, Serum creatinine; BUN, blood urea nitrogen; eGFR, estimated glomerular filtration rate; ACR, albumin-to-creatinine ratio; RRT, renal replacement therapy.

*P < 0.05 versus renal recovery group.

**Supplementary Table 2. Urinary biomarker values grouped according to the renal recovery status**

| **Variables** | **Total** | **Renal recovery** | **Renal non-recovery** | ***P* value** |
| --- | --- | --- | --- | --- |
| uNAG/Cr, U/g Cr | 47.6（25.1, 75.4） | 46.59 (25.76, 69.99) | 50.62 (23.91, 95.42) | 0.295 |
| uTIMP-2•IGFBP7/Cr,  (mg/g Cr)^2^/1000 | 20.34 (5.80, 53.92) | 12.13 (3.74, 36.75) | 28.89 (12.00, 70.98)* | 0.001 |
| uDcR2/Cr, ng/g Cr | 287.07 (174.07, 491.69) | 213.60 (131.89, 331.60) | 419.71 (241.94, 621.98)* | <0.001 |

*P < 0.05 versus renal recovery group.

**Supplementary Table 3. Correlation of uDcR2/Cr levels with clinical and pathological parameters**

|  | **Renal non-recovery** | |  | **Renal recovery** | |
| --- | --- | --- | --- | --- | --- |
|  | ***r*** | ***P*** |  | ***r*** | ***P*** |
| **Clinical parameters** |  |  |  |  |  |
| Age | 0.218 | 0.097 |  | 0.020 | 0.861 |
| eGFR | 0.124 | 0.350 |  | 0.032 | 0.779 |
| Uric acid | 0.189 | 0.151 |  | 0.016 | 0.888 |
| BUN | 0.021 | 0.874 |  | 0.016 | 0.888 |
| Cystatin C | 0.174 | 0.264 |  | 0.000 | 1.000 |
| **Pathological scores** |  | |  |  | |
| Acute injury scores | 0.081 | 0.423 |  | 0.000 | 0.996 |
| Chronic injury scores | 0.217 | 0.028 |  | 0.050 | 0.576 |
| Total scores | 0.077 | 0.418 |  | 0.032 | 0.707 |

**Supplementary Table 4-A. Urinary biomarkers for predicting renal non-recovery from AKI**

| **Variable** | **Cut-off value** | **Sensitivity** | **Specificity** | **AUC (95% CI)** |
| --- | --- | --- | --- | --- |
| uDcR2/Cr | 378.0 | 60.3% | 82.9% | 0.733 (0.646–0.820) |
| uTIMP-2•IGFBP7/Cr | 14.3 | 74.1% | 44.7% | 0.659 (0.566-0.752) |

**Supplementary Table 4-B. Urinary biomarkers and combination models for predicting renal non-recovery from AKI**

| **Variable** | **Cut-off value** | **Sensitivity** | **Specificity** | **AUC (95% CI)** |
| --- | --- | --- | --- | --- |
| eGFR | 378.0 | 60.3% | 82.9% | 0.649 (0.556–0.742) |
| eGFR + total scores | 0.379 | 78.0% | 61.8% | 0.753 (0.671-0.834) |
| eGFR + uDcR2/Cr | 0.345 | 86.4% | 55.3% | 0.762 (0.682-0.841) |
| eGFR + total scores + uDcR2/Cr | 0.366 | 86.4% | 67.1% | 0.824 (0.752-0.895) |

**Supplementary Table 5. Univariable and multivariable logistic regression analyses for risk factors for renal non-recovery**

| **Variable** | **Univariable** | | **Multivariable** | |
| --- | --- | --- | --- | --- |
|  | **OR (95% CI)** | ***P* value** | **OR (95% CI)** | ***P* value** |
| Diabetes | 3.365 (1.196 – 9.468) | 0.021 |  |  |
| CKD | 2.386 (1.146 – 4.971) | 0.020 |  |  |
| eGFR | 0.975 (0.960 – 0.991) | 0.002 |  |  |
| Cystatin C | 1.758 (1.252 – 2.468) | 0.001 |  |  |
| uTIMP-2•IGFBP7/Cr ≥ 14.30(mg/g Cr)^2^/1000 | 3.522 (1.684 – 7.366) | 0.001 |  |  |
| DcR2/Cr, ≥ 378ng/g Cr | 6.667 (3.064 – 14.504) | <0.001 | 8.177 (3.000 – 22.282) | <0.001 |
| Acute injury scores | 1.847 (1.304 – 2.617) | 0.001 |  |  |
| Chronic injury scores | 2.643 (1.738 – 4.018) | <0.001 | 2.143 (1.284-3.574) | 0.004 |
| Total scores | 1.666 (1.331 – 2.085) | <0.001 |  |  |

**Supplementary Table 6. Predictive factors for renal non-recovery by** **univariate and multivariate Cox regression**

|  | **Univariate Cox** | | **Multivariate Cox** | |
| --- | --- | --- | --- | --- |
|  | HR (95%) | *P* value | HR (95%) | *P* value |
| History of diabetes | 2.409 (1.289 – 4.505) | 0.006 |  |  |
| eGFR，mL/min/1.73m2 | 0.983 (0.971 – 0.995) | 0.007 |  |  |
| Cystatin C | 1.228 (1.055 – 1.429) | 0.008 |  |  |
| uTIMP-2•IGFBP7/Cr≥14.30(mg/g Cr)2/1000 | 2.307 (1.130 – 3.673) | 0.005 |  |  |
| uDcR2/Cr (≥378ng/g Cr) | 2.659 (1.575 – 4.488) | ＜0.001 | 3.164 (1.648 – 6.037) | 0.001 |
| Acute injury scores | 1.575 (1.251 – 1.985) | ＜0.001 |  |  |
| Chronic injury scores | 2.112 (1.682 – 2.652) | ＜0.001 | 2.121 (1.531 – 2.938) | ＜0.001 |
| Total scores | 1.491 (1.305 – 1.704) | ＜0.001 |  |  |
